# Possible Role of Autoimmunity in Patients with Premature Ovarian Insufficiency

**Published:** 2013-12-22

**Authors:** Renata Košir Pogačnik, Helena Meden Vrtovec, Alenka Vizjak, Alenka Uršula Levičnik, Nina Slabe, Alojz Ihan

**Affiliations:** 1 Department of Obstetrics and Gynecology, University Medical Centre Ljubljana, Ljubljana, Slovenia; 2 Institute of Pathology, Faculty of Medicine, University of Ljubljana, Ljubljana, Slovenia; 3 Institute of Microbiology and Immunology, Faculty of Medicine, University of Ljubljana, Ljubljana, Slovenia

**Keywords:** Autoantibody, Thyroid Stimulating Antibody, Cell Immunity, Premature Ovarian Insufficiency, T-Lymphocyte

## Abstract

**Background:**

To evaluate the involvement of immune abnormality in patients with idiopathic premature ovarian insufficiency (POI). In addition to the known etiology, autoimmune disorders may be a pathologic mechanism for POI.

**Materials and Methods:**

Our study was a prospective controlled trial. Twenty women with POI, reasons other than autoimmune excluded, were enrolled in this study. The control group consisted of 17 healthy women. In both groups, family and personal history were taken and the levels of follicle stimulating hormone, luteinizing hormone, thyroid-stimulating hormone, prolactin, anti-Müllerian hormone, inhibin B, antithyroglobulin and antithyroid peroxidase antibodies were determined. Antiovarian antibodies and subpopulations of peripheral blood T-lymhocytes were also determined.

**Results:**

Participants in the study group exhibited hypergonadotropichypogonadism, while high levels of follicle stimulating hormone and low levels of inhibin B and anti-Müllerian hormone were observed. In 16 (80%) patients, POI was associated in their personal and familial history with another autoimmune disease. Fifty percent of patients presented highly elevated antithyroid antibodies. The lymphocyte subset, especially B cells, was significantly higher (p=0.014), and peripheral regulatory lymphocytes CD25+ high were significantly lower (p=0.015) in the study group than in the control group. Anti- ovarian antibodies were detected in 20% of patients with POI.

**Conclusion:**

We presume that the presence of anti-ovarian antibodies together with abnormalities of cellular immunity may in some cases potentially represent the involvement of an autoimmune mechanism in idiopathic POI.

## Introduction

Premature ovarian insufficiency (POI) is characterized by hypergonadotropic amenorrhea due to cessation of ovarian function before the age of 40 years. The diagnosis is based on amenorrhea before the age of 40 associated with follicle stimulating hormone (FSH) levels >40 IU/l, detected on two occasions at least one month apart (1). POI causes female infertility, while is a significant psychosocial burden and a risk to women’s health. It occurs in 1% of women, of whom 10-28% have primary and 4-18% secondary amenorrhea (2, 3).

Although there are multiple etiologies of POI (genetic, chromosomal, infectious, and iatrogenic causes), the etiology cannot be identified in most patients and this is referred to as idiopathic POI; up to 30% of idiopathic cases may have an autoimmune cause (4). The most convincing evidence coming from the commonly observed association of POI with other autoimmune disorders (5, 6) are demonstration of anti-ovarian antibodies (AOA, 7, 8) and histological findings of ovarian tissue from affected women. Roughly, one third of POI patients have AOA and/or antithyroid antibodies in their serum (1, 9). Various organ-specific and systemic autoimmune diseases cause autoimmune ovarian insufficiency in up to 30% of women with POI (4). According to the literature, 2-10% of POI cases are known to be associated with adrenal autoimmunity (10). One of the first signs that autoimmunity may be responsible for ovarian function failure came from the observation that ovarian failure may precede the onset of Addison’s disease by 8-14 years (11). Autoimmune Addison’s disease seldom develops in isolation, whereas several other endocrine glands and organs are generally affected, leading to an autoimmune polyglandular syndrome (APGS, 12). Two main forms of APGS can be clinically discerned, APGS types 1 and 2. APGS type 1 is characterized by an association of mucocutaneous candidiasis, hypoparathyroidism and Addison’s disease. In about 60% of cases, there is also an association with ovarian insufficiency. Blizzard et al. (13) and Irvine et al. (14) found that POI commonly presents with adrenal cytoplasmic antibodies, called steroid cell antibodies (SCA); they react with cytoplasmic antigens of other steroid-producing cells present in the ovary, testis and placenta. Alteration of lymphocytes and their specific subsets, as well as T-cell mediated injury are likely to play an important role in the pathogenesis of autoimmune oophoritis. Surface markers of peripheral blood mononuclear cells (PBMC) have been shown to be deranged in early autoimmune phases and to be persisted through the disease, even after targeted disruption (15).

The presence of pathogenic factors might accelerate the process of apoptosis and atresia of ovarian follicles during the fetal and post-natal period (16). This interpretation is based on the dogma that the number of ovarian follicles at birth is final and that there is no possibility of regeneration or renewal of reserve follicles in adulthood (17). Experimental work on animals suggests a possibility of renewal of the follicle reserve from proliferative germinal ovarian cells even after birth; verification of which is being sought in studies on human ovaries (18, 19). It has been shown that undifferentiated ovarian stem cells differentiate into structures similar to egg cells under certain laboratory conditions (20). We faced two problems: whether to accept the standard understanding of a final number of ovarian follicles or to focus on the hypothetic possibility of renewal of the follicular reserve. The aim of this study was to evaluate the involvement of immune abnormality in patients with idiopathic POI.

## Materials and Methods

### Subjects

Our study was a prospective randomized controlled trial. The study group consisted of 20 women with POI (mean age 31.8 years, range 20-39 years) and no use of medications or oral contraceptives for at least 4 months prior to the study. The diagnosis was based on the presence of amenorrhea before the age of 40, associated with two serum FSH levels above 40 IU/l at least one month apart. All women with POI underwent karyotyping and genetic testing of the fragile X mental retardation 1 (FMR1). Women with infectious or iatrogenic causes were excluded from the study. The control group consisted of 17 healthy women volunteers. Inclusion criteria were a regular menstrual cycle, age between 18 and 39 years (mean 30.8 years), and no use of medications or oral contraceptives for at least 4 months prior to the study. They also had to be exempt from autoimmune disease or infertility problems. All women provided a complete personal and family history, with a stress on possible immune-mediated, particularly autoimmune processes (allergy, asthma, diabetes, thyroiditis, rheumatoid arthritis, andatopiceczema), and all underwent physical and vaginal ultrasound examinations.

### Hormone and serological analyses

Serum levels of luteinizing hormone (LH), FSH, estradiol (E_2_), prolactin (PRL), inhibin B, thyroid- stimulating hormone (TSH), anti-Müllerian hormone (AMH), antithyroglobulin (aTG) and antithyroid peroxidase antibodies (aTPO) were measured, and immunological investigations at cellular and humoral levels were performed. The adrenocorticotropic hormone (ACTH) stimulation test was performed in the study group, only. The standard Synacthen stimulation test is clinically widely used as a sensitive screening method for symptomatic adrenal insufficiency. Each ampoule of Synacthen contains 250 μg of the active ingredient, tetracosactrin (Novartis Pharmaceuticals, North Ryde NSW, Australia). Thirty minutes after 250 μg Synacthen I.M. (Alliance Pharmaceutical Wiltshire, UK), blood cortisol was measured by electro-chemiluminescence immunoassay. A normal cortisol response to Synacthen was defined as a post-stimulation peak cortisol value of >500 nmol/l at 30 minutes.

Serum AMH in peripheral blood was determined by a Personal Lab analyser using the enzyme linked immunosorbent assay (ELISA) method with Beckman Coulter reagent. The normal range of AMH levels is 0.7-3.5 mg/l. Values below 0.3 mg/l indicate a reduced ovarian reserve. Hormones were determined on a LIAISON analyser by quantitative direct competitive chemiluminescence immunoassays (CLIA). Each test is a modified two-step process, in which the specific antibodies of a certain hormone bind to magnetic cells. Normal ranges for the follicular phase of the cycle are as follows: FSH: 3.5-9.2 IU/l; LH: 1.1-11.6 IU/l; LH around ovulation: 17-77 IU/l; PRL: 6.2-23.5 μg/l; E_2_: on day 3 up to 310 pmol/L (0.31 nmol/L); E_2_ postmenopause: 0-110 pmol/L (0-0.11 nmol/l); and TSH: 0.3-3.6 mE/l. Serum Inhibin-B cut-off level on day 3 was 45 pg/mL. Serum aTG and aTPO concentrations measured by immunoassay using direct chemiluminometric technology on an ADVIA CENTAUR analyser (Siemens Medical Solutions Diagnostics, Tarrytown, USA). The normal range for serum aTG and aTPO is <60 KE/l. Detection of AOA was by indirect immunofluorescence (IIF) on cryosections of normal human ovarian tissue.

Non-human primate ovaries for the detection of AOA are not commercially available, while normal human ovarian tissue is available at the Institute of Pathology, Ljubljana, Slovenia, when ovaries are removed due to various pathological processes, particularly tumors, and are sent for pathohistological examination. Ovarian tissue was incubated with patient serum diluted 1:10. The second incubation was with fluorescein isothiocyanate-labelled anti-human IgG antibody (Dakopatts, Copenhagen, Denmark). Positive reactions were semi-quantitatively evaluated (on a scale of 1– 4+). Negative control omitting the patient’s serum was regularly included (21). At the cellular level fresh PBMC were studied by flow cytometry (Becton Dickinson FACS, NJ, USA), and percentages of the following blood lymphocyte populations were determined: T cells (CD3+), helper T cells (CD4+), cytotoxic T cells (CD8+), natural killer cells (CD56+CD16+), regulatory T cells (CD25^+high^) and B cells (CD19+) (22). Immunofluorescence labelling was performed by incubating PBMCs with monoclonal antibodies to CD3, CD4, CD8, CD25, CD56/16 and CD45. A differential blood count with a standard laboratory procedure was taken to obtain concentrations of individual lymphocyte subtypes.

### Statistical analysis

Normal data distribution was tested with the Kolmogorov-Smirnov test. Where variables were normally distributed, we used the Pearson’s chisquare test. If variables were not normally distributed, we used a nonparametric Mann-Whitney test. Statistical analysis was done using Statistical Package for the Social Sciences, version 18 (SPSS Inc., Chicago, IL, USA). The results were considered statistically significant at p<0.05.

### Ethical considerations

The study protocol was approved by the National Ethics Committee, and all patients gave written informed consent.

## Results

### General clinical and biological parameters

The subjects were comparable with controls by age. One patient presenting a mosaic 45X0/46XX was excluded from the final analysis. Other patients had a 46XX karyotype. Anamnestic data of patients showed that four had had mumps during their childhood; they were thus excluded from the final analysis. Table 1 shows the clinical and endocrine characteristics of patients and of healthy women. All endocrine parameters in controls were within the normal range (Table 1).

**Table 1:** Hormone and ovarian peptide levels in patients and in healthy controls


Mean value+/- SD	Study group (n=20)	Control group (n=17)

**FSH (IU/L)**	88.04 +/- 47.93	6.25 +/- 2,77
**LH (IU/L)**	37.85 +/- 18.71	11.64 +/- 31.1
**E_2_ (pmol/L)**	180 +/- 200 (0.18 +/- 0.2nmol/L)	210+/- 130 (0.21 +/- 0.13nmol/L)
**PRL (μg/L)**	11.14 +/- 6.42	8.74 +/- 5.51
**Inhibin B (pg/ml)**	13.36 +/- 10.66	32.63 +/- 24.05
**AMH (mg/L)**	0.36 +/- 0.37	3.54 +/- 1.58


POI; Premature ovarian insufficiency, FSH; Follicle stimulating hormone, LH; Luteinizing hormone, E_2_; Estradiol, PRL; Prolactine and AMH; Anti-Müllerian hormone.

### Prevalence of autoimmune diseases and associated autoimmune abnormalities

We collected targeted history information on personal and familial autoimmune disorders (Table 2). Sixteen patients (80%) had an associated autoimmune disease in their personal and/or familial history. Four women (20%) with POI had first grade relatives with ovarian insufficiency before the age of 40. Thyroid disorders were the most common (15%) of the autoimmune diseases associated with POI in personal histories. Before entering the study, three patients had been treated for autoimmune thyroid dysfunction (Hashimoto thyroiditis). In the study group, 55% of women had autoimmune disease in the family history; the most frequent autoimmune disorder was diabetes type I. The overall personal and familial incidence of autoimmune diseases was lower in the control group. In the study group, 50% of women had aTG. One healthy woman had evidence of autoimmune thyroid dysfunction, manifested by an elevated aTG serum level, and was excluded from the final analysis (Tables 2,3).

**Table 2:** Personal and family history on autoimmune diseases in patients and healthy controls


	Study group (n=20)		Control group (n=17)	
	Family history	Personal history	Family history	Personal history

**Diabetes type I**	7		1	
**Psoriasis**	2	2	1	1
**Rheumatism**	2			
**Thyroid disease**		3	2	1
**Vitiligo**	1	1		
**POI**	4			
**Atopic dermatitis**		1		1


POI ; Premature ovarian insufficiency.

**Table 3:** Associated autoimmune abnormalities (some participants showed more than one condition)


Associated autoimmune findings	Study group	Control group

**Anti-thyroglobulin antibody (aTG)**	10 (50%)	0
**Positive ACTH test**	5 (25%)	0
**Anti-ovarian antibodies (AOA)**	4 (20%)	0
**Personal history of autoimmune disease**	6 (30%)	2 (11.1%)
**Family history of autoimmune disease**	11 (55%)	4 (22.2%)
**POI in family members**	4 (20%)	0


POI; premature ovarian insufficiency.

### Anti-ovarian antibodies

**Fig 1:**
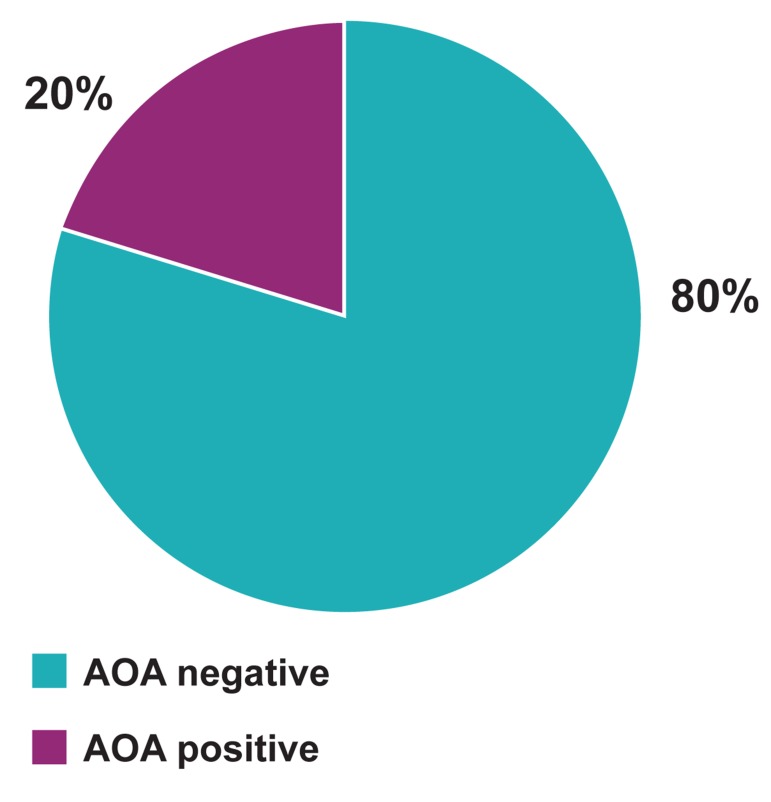
Prevalence of serum anti-ovarian antibodies (AOA) in patients.

Analysis of AOA in serum was performed for all patients (Fig 1). AOA were detected in 4 (20%) patients, and in 3 patients, we found an associated autoimmune disease; Hashimoto thyroiditis. There was a clear positive immune fluorescent reaction of AOA in the serum of one woman who had mumps during her childhood, and she was excluded from the analysis. AOA were not detected in any of the controls (Table 3).

### Lymphocyte subtypes

Cellular autoimmune reaction occurring due to a changed T cell function was analysed as a potential cause of POI. Cell abnormalities were more frequent in women with POI than in healthy women (Table 4).

Peripheral T cell count is expressed as a percentage of various cell surface markers. In patients with POI, peripheral regulatory T lymphocytes (CD25^+high^, p=0.015) were low and peripheral blood B cells (CD19+) were high (p=0.014); T lymphocyte parameters were normal in the control group.

**Table 4:** Prevalence (in %) of analysed peripheral blood lymphocyte samples for various cell surface markers in patients and in controls


Various cell surface markers	Study group (%)	Control group (%)	P value

**CD3^+^**	73.25	74.53	0.578
**CD19^+^**	11.90	9.41	0.014
**CD4^+^**	47.45	45.82	0.450
**CD8^+^**	25.85	28.53	0.229
**CD25^+^^h^^i^^g^^h^**	1.60	2.85	0.015
**CD56^+^CD16^+^**	14.20	16.23	0.366


Markers for peripheral blood lymphocytes: T lymphocytes (CD3+), helper T lymphocytes (CD4+), cytotoxic T lymphocytes (CD8+), natural killer cells (CD56+CD16+), regulatory T lymphocytes (CD25^+high^) and B cells (CD19+).

## Discussion

To present POI as a possible autoimmune abnormality, we focused on three potentially interconnected factors: personal and familial history of autoimmune disorders, peripheral blood T-lymphocytes levels and presence of AOA.

We performed a targeted history of personal and familial autoimmune disorders and found associated autoimmune disorders in our patients. Thyroid disorders were common in personal histories and diabetes type 1 in familial histories, confirming the findings of previous studies (5, 6, 15). One of the reasons for suspecting an autoimmune etiology of POI is its frequent association with nearly all organ-specific autoimmune diseases (1, 3). Autoimmune diseases are significantly more frequent in young women than in men. This phenomenon may be explained by the effect of sex steroids on the components of the cellular immune system, which might contribute to the development and progression of autoimmune POI (23).

Hoek et al. (24) found that 60% of patients with secondary amenorrhea and Addison’s disease have a detectable SCA serum titre. These antibodies are shown by 60-80% of patients with APGS type I. In 25% of our patients, the Synacthen test revealed an abnormal cortisol response. We interpreted this as the possibility that patients could be positive for SCA, but had a normal cortisol response. Patients with this disorder have an insidious onset; to confirm subclinical autoimmune adrenal insufficiency, measurement of adrenal antibodies might be a more effective screening method (25, 26).

Thyroid disorders and the presence of antithyroid antibodies are often mentioned in association with POI. Thyroid disorders are the most common of the autoimmune diseases associated with POI, found in 12-39% of women with POI (27- 29). Thyroid disorders are often associated with endometriosis and polycystic ovary syndrome, two conditions often resulting in infertility (2). We found a greater involvement of aTG and aTPO in the study than in the control group, and the explanation being that ovarian failure may have been present in the latent period of thyroid disease. We found a strong correlation between autoimmune thyroid disease and autoimmune POI; 50% of patients tested positive for aTG. We concluded that not all patients with positive aTG necessarily have a clinically expressed thyroid disorder and the disease can have an insidious onset. Furthermore, greater involvement of other immune-mediated diseases, particularly auto immune disorders, such as allergy, psoriasis, atopic dermatitis and vitiligo in the study group suggests an autoimmune cause of POI, consistent also with the findings in the literature (9, 15, 30, 31).

Genetic predisposition is known to be one of the primary causes of autoimmunity and it is generally observed that patients with autoimmune diseases have several types of antibodies, as also confirmed in our study. We found AOA in 20% of patients; these results are consistent with other studies (9, 21, 32-35). The prevalence of AOA in women with POI, studied with IF on ovary tissue, varies greatly from 2 to 70%, which supports their role as a marker of an immune dysfunction process against ovaries. There could be several reasons for the differences among study results, including the diverse origin of tissue sections, which include human and non-human primate ovaries, as well as different study inclusion criteria. Many studies have shown that the presence of serum AOA does not correlate with the clinical manifestation of POI. Despite these antibodies being present, their pathogenetic role is highly questionable (36, 37). AOA may occur several years before the occurrence of clinical symptoms, as detected in 33-61% of women with unexplained infertility; a situation that may indicate early stages of autoimmune ovarian insufficiency (38, 39). For most autoimmune diseases, screening for specific antibodies is probably the best way of evaluating immunological involvement. Many ovarian structures are potential targets for autoimmune events with POI. In addition to AOA, SCA, aTG and aTPO, antibodies to several potential ovarian antigens have been proposed as markers of ovarian autoimmunity, which could potentially mediate autoimmune damage in POI. Betterle et al. (40) found antibodies against steroid genetic enzymes in some cases with anti-adrenal autoimmunity. Antibodies against gonadotropin receptors and gonadotropins have also been found in patients with POI, but they are still the subject of research. The general opinion is that more research is needed (41, 42). Although some studies have managed to identify autoantibodies against zona pellucida, corpus luteum and ovarian cells, these findings have no real correlation with the clinical picture (43-45). In our preliminary study, AOA were determined semi-quantitatively by the IIF method. IIF is a basic method and widely used for determining auto antibodies, while more specific tests enabling detection of AOA for specific antigen targets are not available in our laboratory.

Infertile women with AOA also have a decreased response to gonadotropin stimulation and reduced pregnancy rate after treatment (46, 47). In 2002, it was found that low-responders to gonadotropins with AOA are younger than low-responders without AOA (48). Detection of autoimmune processes that affect the ovarian response should thus be included in the diagnostic workup before any infertility treatment, particularly in women with low or no response to gonadotropins (34). Determination of AOA, as a specific test, is important in the diagnosis of diseases with an autoimmune etiology, but it should not be the only reliable diagnostic tool for optimal selection of patients who may benefit from immune modulatory therapy that could, at least temporarily, re-establish their ovarian function and fertility.

Abnormalities of cellular immunity of T lymphocytes, macrophages and dendritic cells play an important role in autoimmune events. Some of these abnormalities have been seen in women with POI, confirming the potential existence of an autoimmune mechanism of the disease. Mignot et al. (49) found that the absolute number and percentage of peripheral blood T lymphocytes, especially CD4+ T cells, are increased in patients with POI. Moreover, Miyake et al. (50) found that patients with POI have low levels of CD8+/CD57+ Tcells (cytotoxic T lymphocytes) and an increased ratio of CD4+ to CD8+ cells, which may reflect cell cytotoxic cell migration from blood to inflamed tissue. Multiple animal studies have suggested that the basis of POI is a cell-mediated autoimmune reaction caused by an alteration in T cell regulation (18). CD4+ T cells with constantly expressed α chain (CD25) receptor for IL-2 of were the first detected mediators of inhibition of autoimmune diseases in mice alteration of suppressor T cell subsets and T cell abnormalities are likely to play an important role in the pathogenesis of autoimmune diseases (51). Regulatory CD4+25+ T cells show a potent immunosuppressive function in vitro and in vivo, and contribute to immunologic self-tolerance by suppressing potentially auto-reactive CD4+ T cells. There is known to be a number of immunological mechanisms associated with the failure of immune tolerance and the development of autoimmunity. Although studying regulatory T cells in human autoimmune diseases is difficult, and at times, findings have been contradictory, the data suggest that defects in CD4+ CD25+ regulatory T cells mediated suppression (52) are a major subset of immune cells responsible for peripheral immune self-tolerance.

In agreement with studies in patients with systemic lupus erythematosus, multiple sclerosis, rheumatoid arthritis and autoimmune vasculitis, we confirmed a reduced number of CD4+CD25^+high^ T cells in the peripheral blood of our patients. High expressions of CD25 and CD4 surface markers have classically been used for identification of regulatory T cells. This may be problematic since CD25 is also expressed on antigen-responding activated non-regulatory T cells. The additional measurement of cellular expression of Foxp3 protein allowed a more specific analysis of Treg cells (CD4+CD25+Foxp3+ cells). However, Foxp3 is also transiently expressed in activated human effector T cells, thus complicating a correct Treg analysis. The large majority of Foxp3-expressing regulatory T cells express high levels of the interleukin-2 receptor alpha chain (CD25). Since there are no cell surface markers that are uniquely and specifically expressed on all Foxp3-expressing regulatory T cells, the measurement of CD4+CD25^+high^ T cells is still in use in clinical studies of peripheral blood lymphocytes.

We interpreted the reduced number of CD4+CD25^+high^ T cells as a possible mechanism contributing to the formation of an autoimmune response in association with the presence of AOA and aTG. We also found an increased number of B cells in peripheral blood. A similar picture has been observed with other autoimmune endocrinopathies; therefore, we interpreted the elevated B cell count as activation of the humoral immune system, crucial for autoantibody production. Some authors, though, have tried estrogen substitution in women with POI without any effect on peripheral B cell count (53, 54).

The hormones inhibin B, FSH and AMH have been proposed as potential markers for determining the functional ovarian reserve (55, 56). In young ovulatory women, measurement of AMH at 3-year intervals has shown that the AMH serum level decreases significantly over time, whereas other markers associated with ovarian aging, such as FSH, inhibin B and antral follicle count (AFC), do not change during this time period. Since it decreases at a time when concentrations of FSH and inhibin B are still normal, AMH has been proposed as the best indicator of ovarian reserve and as a marker of ovarian aging (55-59). In contrast, AMH is almost undetectable in women with POI (60), which our study also confirmed. In our group of patients, there was a small AFC or these structures were no longer seen on ultrasound, which agrees with the data in the literature (61).

## Conclusion

Clinical and biological characteristics of women without known causes of disease suggest a possibility of autoimmune pathogenesis. In some patients, a combination of various autoimmune processes has been found. The presence of AOA and anti-thyroid antibodies, together with abnormalities of cellular immunity, potentially represent an autoimmune mechanism of POI. There is thus an increasing need to find a reliable and simple diagnostic procedure to determine the true prevalence of autoimmune ovarian disease. In women with POI, more attention should be paid to evaluation of associated autoimmune disorders.
